# Increased Circulating Irisin Levels in Kidney Transplant Patients: Is There a Connection with Glycaemic Control?

**DOI:** 10.3390/ijms25052926

**Published:** 2024-03-02

**Authors:** Beata Bzoma, Agnieszka Kuchta, Kornelia Sałaga-Zaleska, Aleksandra Krzesińska, Gabriela Chyła-Danił, Maciej Jankowski, Alicja Dębska-Ślizień

**Affiliations:** 1 Department of Nephrology, Transplantology and Internal Diseases, Medical University of Gdańsk, Smoluchowskiego 17, 80-214 Gdańsk, Poland; adeb@gumed.edu.pl; 2 Division of Clinical Chemistry, Medical University of Gdańsk, Dębinki 7, 80-211 Gdańsk, Poland; agnieszka.kuchta@gumed.edu.pl (A.K.); kornelia.salaga-zaleska@gumed.edu.pl (K.S.-Z.); aleksandra.krzesinska@gumed.edu.pl (A.K.); gabriela.chyla-danil@gumed.edu.pl (G.C.-D.); maciej.jankowski@gumed.edu.pl (M.J.)

**Keywords:** kidney transplantation, irisin, glycaemic disturbances

## Abstract

Irisin is a myokine with potential effects on glucose metabolism and the development of diabetes in humans. We analysed irisin serum levels (ISL) in 47 patients without diabetes before and 1, 2, 3, 4 and 5 weeks after kidney transplantation (KTx). All measurements of irisin before KTx levels were lower than 25 ng/mL (median 8.4 ng/mL). We found an outstanding increase in ISL measured after KTx, reaching more than 1000 times in 44% of patients (HIL—high irisin level group). The increase appeared at the first measurement (one week after KTx). Factors connected to the large growth of ISL were, i.e., BMI > 30 (*p* = 0.04) and subsequent KTx—second and third (*p* < 0.001). The global mean blood glucose level during the first two weeks after KTx was significantly lower in the HIL group (*p* = 0.002), the same as the day-by-day analysed mean fasting and postprandial serum glucose in the first days after KTx. In 12 months of observation, diabetes requiring insulin therapy occurred in the HIL group at a rate of 19%, while in the rest of the patients, the rate was 27%, *p* = 0.526. Irisin levels increase significantly in some patients after kidney transplantation, accompanied by lower blood glucose levels in the early post-transplant period. Whether an increase in irisin levels results in better glycaemic control remains questionable and requires further research, as well as the relationship between irisin levels and the occurrence of PTDM.

## 1. Introduction

There is increasing evidence that skeletal muscle functions as an internal secretory organ [1]. Irisin is a myokine. It is mainly released from muscles in response to exercise and is widely distributed in the human body, and it binds to a yet undiscovered receptor on the surface of other tissues; however, in some tissues, it probably acts via a specific class of integrin receptors (αV/β5) [2]. Irisin affects the metabolism of the body and the communication between tissues. It was discovered in 2012 and was named after the ancient Greek goddess, the personification of the rainbow and messenger of the gods [3]. Irisin is a glycosylated protein hormone consisting of 112 amino acids, a PGC-1α (proliferator-activated receptor-γ coactivator-1α)-dependent myokine, a fragment of the extracellular domain of the FNDC5 (fibronectin type III domain-containing protein 5) transmembrane protein [3]. Irisin forms a continuous intersubunit β-sheet dimer [4]. The C-terminal fragment of FNDC5 is located in the cytoplasm, whereas the extracellular N-terminal portion is proteolytically cleaved to produce irisin. Irisin is completely conserved among vertebrates and has a 100% similarity between mice and humans [4].

The proposed beneficial effects of irisin include the browning of white adipose tissue and increased thermogenesis, which promotes insulin sensitivity, increased body weight, and glucose tolerance in mice [3,5,6]. Irisin has been proposed to improve glucose homeostasis by increasing fatty acid oxidation and utilising glucose via the AMPK signalling pathway in diabetic mice [7]. In human subjects, elevated circulating irisin levels are associated with a lower risk of insulin resistance in obese adults [8,9]. However, it was also reported that a high irisin level is associated with a higher risk of metabolic syndrome and cardiovascular disease, which could be the result of a compensatory increase in the secretion of irisin by muscle tissue and/or to overcome underlying irisin resistance in metabolically affected patients [10]. 

Glucose tolerance disorders are a common complication in the post-renal transplantation period, and they are characterised by a combination of insulin resistance and insulin hyposecretion. Risk factors for PTDM (post-transplant diabetes mellitus) include both general diabetes risk and some specific to transplant patients, such as immunosuppression. PTDM is associated with an increased risk of renal graft failure, cardiovascular disease and death [11,12,13,14]. A problem in daily practice in transplant centres is acute hyperglycaemia occurring in up to 90% of kidney recipients in the first weeks after transplantation [15,16]; most patients show an improvement in glucose tolerance after reducing the dose of immunosuppressive drugs, although early development of hyperglycaemia is a strong predictor of PTDM [17]. PTDM occurs in about 20–30% of kidney transplant recipients [11,18]. The role of irisin in glucose metabolism and the development of diabetes in humans is controversial. Irisin serum concentrations were not previously analysed in transplant patients and in post-transplant diabetes mellitus (PTDM). We also investigated the possible association between circulating irisin levels and glucose metabolism and diabetes after kidney transplantation. 

## 2. Results

### 2.1. Irisin Serum Levels (ISLs) in Patients before and after KTx in the Studied Group

All measurements of ISL before KTx levels were lower than 25 ng/mL (median 8.4 ng/mL). 

**Figure 1:**
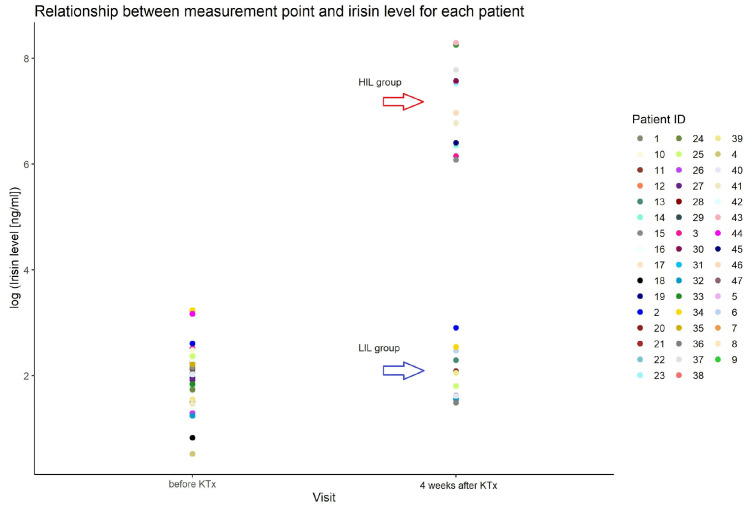
Irisin serum level in patients before and after KTx in studied group.

We found an outstanding increase in ISL measured after KTx, reaching more than 1000 times in 44% of patients (21/47) (HIL). The increase appeared at the first measurement (one week after KTx) and remained stable for at least a month. The median value of all measurements after the KTx procedure in the group with an increase in ISL was 1220 ng/mL (min–max: 158–5884), and in the group without irisin increase (LIL), it was 6 ng/mL (min–max: 2–20) (Figure 1).

### 2.2. Clinical and Biochemical Factors Analysed Comparatively in HIL and LIL of Patients after KTx 

All the factors analysed are listed in Table 1.

#### 2.2.1. Factors That Are Statistically Significantly Associated with a Large Growth in ISL

a. Obesity

In the HIL group, there were more obese patients with BMI > 30 kg/m^2^ (*p* = 0.04).

b. Duration of renal replacement therapy and subsequent KTx—second and third

The duration of renal replacement therapy (total time spent on dialysis and transplantation, counted from the first dialysis) was longer in the HIL group, with 81.5 months in the HIL group and 26.0 months in the LIL group (*p* = 0.005). 

Subsequent KTx—second and third—were connected to the large growth in ISL (*p* < 0.001). There were no patients in the LIL group after subsequent KTx.

c. Cold ischaemia time

The cold ischaemic time of the transplanted kidney was longer in the HIL group (*p* = 0.05).

d. Immunosuppression

Tacrolimus was part of immunosuppressive treatment in 89.4% of all patients; the rest of them received cyclosporin (10.6%). Tacrolimus was used equally often in both groups (HIL and LIL). The mean tacrolimus blood level on the 6th day after KTx was statistically significantly higher in patients in the HIL group, *p* = 0.001. 

There was a positive correlation between tacrolimus and glycaemia levels by the analysis on the 6th day after KTx for fasting glucose (r(df) = 0.28; *p* = 0.05) and for postprandial glucose (r(df) = 0.05; *p* = 0.05).

In patients who received cyclosporin, its levels did not differ between the HIL and LIL groups.

All patients received only initial boluses of methylprednisolone within the first 3–4 days after KTx, followed by standard oral therapy. No patient in the entire study group had a diagnosis of acute rejection of the transplanted kidney, so none of the patients received additional doses of intravenous steroids.

The two-week cumulative dose of prednisone per kg body weight was 4.8 ± 1.1 mg/kg in the entire study group. The cumulative dose of prednisone was 4.68 ± 0.9 and 4.97 ± 1.2 mg/kg in the LIL and HIL groups, respectively (*p* = 0.36).

#### 2.2.2. Factors Not Statistically Significant in the Comparison of HIL and LIL Groups

We did not find an influence of gender, age, cause of ESRD, dialysis modality before KTx or delayed graft function (DGF, defined as the need for dialysis during the first week after KTx) related to the increase in the irisin level. 

The HIL and LIL groups did not differ in terms of kidney graft function one month after KTx, pre-transplant HbA1c, glucose, C-peptide, insulin, HOMA index, cholesterol and acute rejection (AR) in both groups (Table 1). 

#### 2.2.3. Glycaemic Disturbances in the Studied Group of Patients in the Time soon after KTx 

The blood glucose analysis in patients after KTx in studied groups: HIL and LIL

Global mean blood glucose level during the first two weeks after kidney transplantation was statistically significantly higher in the LIL group (147.2 vs. 127.5 mg/dL, *p* = 0.002). Global mean blood glucose was calculated from all blood glucose measurements determined three times a day during hospitalisation for 2 weeks after kidney transplantation.

Glycaemic values above 200 mg/dL occurred in 8.9% of measurements in patients in the first two weeks after kidney transplantation in the HIL group, and this was statistically significantly less than in the LIL group (*p* = 0.007); in this group, 13.6% of measurements were above 200 mg/dL. 

**Figure 2:**
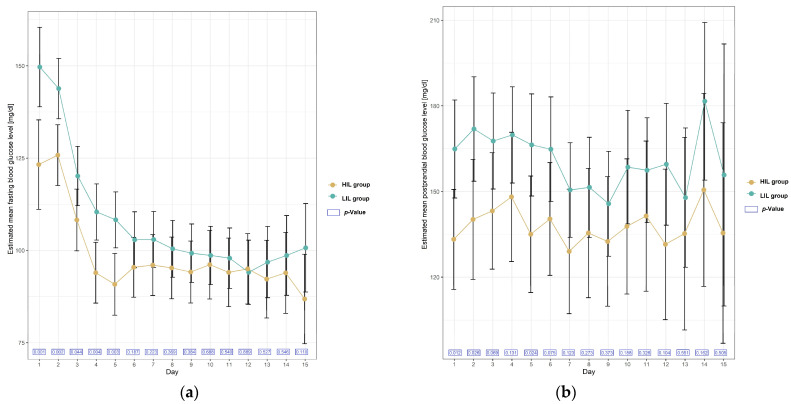
Plots of estimated mean with 95% confidence interval in blood glucose level [mg/dL] in patients with and without irisin increase (HIL and LIL group) during 15 days after KTx. (**a**) Mean fasting blood glucose level; (**b**) mean postprandial blood glucose level.

A day-by-day analysis of blood glucose levels showed that the mean fasting blood glucose level was higher almost every day in the LIL group, but the relationship was statistically significant on days 1–5 (Table 2, Figure 2).

A day-by-day analysis of postprandial blood glucose levels showed that mean glucose was also higher each day in the LIL group, and the relationship was statistically significant on days 1, 2 and 5 (Table 3, Figure 2).

#### 2.2.4. Diabetes Requiring Permanent Insulin Therapy (DM) 

In the entire study group, glycaemic disturbances requiring permanent insulin therapy (DM), defined as the need for insulin therapy more than 3 months after KTx and continuing, occurred in 11 people (11/47)—23%. In the HIL group, DM occurred at a rate of 19% (4/21), while in the LIL group, it was 27% (7/26)—a difference not statistically significant (*p* = 0.526). All of these patients required insulin therapy at checkpoints 3 months and 12 months after kidney transplantation. 

There was one more patient, aged 63, in the LIL group, who required pharmacotherapy; he was taking an oral diabetes treatment—metformin. The addition of this case does not change the result that there was no difference in the frequency of DM in the LIL and HIL groups, *p* = 0.36).

Gender, cause of end-stage renal disease (ESRD), dialysis method used before KTx, DGF, type of calcineurin inhibitor (tacrolimus or cyclosporin), glycaemia, HOMA and Quicki index, insulin level before KTx and BMI did not predispose to the development of DM. In the group of DM, HBA1c before transplantation was significantly higher (*p* = 0.021), as well as total cholesterol (*p* = 0.011) and LDL cholesterol levels (*p* = 0.017); these patients were also significantly older (58 vs. 45 years old (*p* = 0.05)).

#### 2.2.5. Blood Pressure in the Studied Group of Patients after KTx

**Figure 3:**
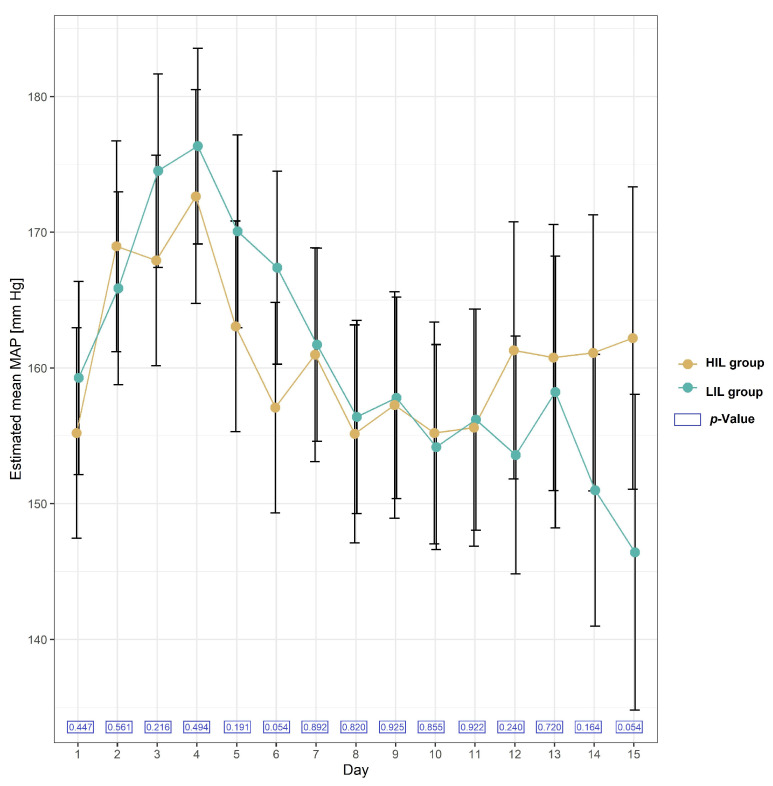
Plot of estimated mean with 95% confidence interval in MAP (Mean Arterial Pressure) [mmHg] in patients with and without irisin increase (HIL and LIL group) during 15 days after KTx.

The mean values of Mean Arterial Pressure (MAP) were not significantly different in the HIL and LIL groups. The formula used to calculate MAP was as follows: MAP = DP + 1/3 (SP − DP), where DP is the diastolic blood pressure, and SP is the systolic blood pressure. The mean values of MAP on each day during the two-week follow-up after KTx in the HIL and LIL groups are presented in Figure 3.

#### 2.2.6. Obesity and Irisin Serum Levels 

Irisin levels tested before kidney transplantation did not differ between patients with BMI above and below 30 kg/m^2^ (mean 9 vs. 8 ng/mL, *p* = 0.64). 

Irisin levels measured one (mean 801 vs. 5303 ng/mL, *p* = 0.01), two (median 30 vs. 48, *p* = 0.06), three (mean 351 vs. 2793 ng/mL, *p* = 0.001) and four weeks after kidney transplantation (mean 547 vs. 1451; *p* = 0.12) were higher in the group with BMI above 30 kg/m^2^.

## 3. Discussion

The most important observation in our study is the surprisingly high increase in irisin levels, reaching more than 1000 times in some patients in response to the kidney transplant procedure and immunosuppression. 

All measurements of ISL before KTx were lower than 25 ng/mL (median 8.4 ng/mL). Lower irisin concentrations have been previously observed in chronic kidney disease (CKD) patients [17,19,20]. The serum irisin level of patients in CKD stage 4 was significantly reduced compared with CKD stage 2 patients [21]. The lowest irisin concentrations were observed in patients with ESRD [19], and furthermore, irisin levels were lower in dialysis patients than in nondialysis patients and lower than in healthy controls [22]. Irisin is produced within muscles, and total muscle volume can affect the irisin level with the progression of kidney failure; a gradual decrease in muscle mass is observed, and it is one possible mechanism of the reduction of the irisin level. 

Also, uremic toxins can contribute to decreased serum concentration of irisin in higher CKD stages. On the other hand, inflammation, oxidative stress, and activation of advanced protein glycation routes may be the causes of irisin reduction in patients with CKD [10,23]. Additionally, irisin is, in part, dialyzable; median irisin levels significantly decreased by 23% after haemodialysis as compared with predialysis concentrations [20]. In our material, irisin levels before transplantation were low in each group: peritoneal dialysis, haemodialysis and pre-emptive transplantation (without prior dialysis). We also did not find the influence of the DGF on the increase in the irisin level. 

Before kidney transplantation, all patients, regardless of BMI (min–max, 18–34), had low levels of irisin in their serum. Only the transplantation procedure and the initiation of immunosuppressive treatment resulted in an outstanding increase in irisin levels, reaching more than 1000 times in 44% of patients (HIL group). Interestingly, surgery alone, for example, bariatric surgery, was not associated with a change in serum irisin levels [24,25].

This is the first report of that kind in post-transplant patients. The increase in irisine appeared at the first measurement (one week after KTx) and remained stable for at least a month. 

In the HIL group, there were statistically significantly more people with BMI > 30. It remains questionable whether body composition, with BMI, muscle mass and fat mass as determinants of the nutritional status of patients with ESRD, influenced the large increase in serum irisin concentration after transplantation in the study group.

An interesting observation is that every patient who underwent a second or third KTx was characterised by a large increase in irisin. It is not surprising that the time these patients remained on renal replacement therapy (total time spent on dialysis and transplantation counted from the first dialysis) was longer—27.6 vs. 165.3 months—and included long periods of diabetogenic treatment—steroids and calcineurin inhibitors. On the other hand, in our study, second and third transplants did not predispose to the development of glycaemic disturbances requiring permanent insulin therapy. The group with and without irisin increase did not differ in the schema of immunosuppression, use of the type of calcineurin inhibitor or induction therapy (application of polyclonal or monoclonal antibodies). No patient in the entire study group had a diagnosis of acute rejection of the transplanted kidney, so all patients received only initial boluses of methylprednisolone within the first 3–4 days after KTx, followed by standard oral therapy. 

In our study, the global mean blood glucose level during the first two weeks after KTx was statistically significantly higher in the LIL group. The mean fasting and postprandial blood glucose levels were higher almost every day in the LIL group; the relationship was statistically significant on days 1–5, on the days of the highest exposure to diabetogenic immunosuppressants, in particular boluses of methylprednisolone. In the following days, however, the relationship remained the same; the mean glucose concentration in the daily analysis was higher in the LIL group, although without statistical significance, probably due to the smaller number of observations, which is a limitation of the study. Whether an increase in irisin could be one of the mechanisms for managing post-transplant hyperglycaemia in renal transplant patients remains questionable. Post-transplant hyperglycaemia has a multifactorial aetiology and may be influenced by tacrolimus exposure. Tacrolimus results in not only insulin secretion deficiency but also insulin resistance. The mean tacrolimus level on day 6 after KTx was higher in the LIL group in our study. Thus, exposure to tacrolimus may be an interfering factor with glucose levels and interferes with the deduction of the effect of irisin, which is also a limitation of this study. On the other hand, it should be mentioned that although borderline significant, the correlation between tacrolimus levels and glycaemia on day 6th after KTx was weak. 

In our study group, glycaemic disturbances requiring permanent insulin therapy (DM), defined as the need for insulin therapy 3 months after KTx and 12 months after KTx, were noticed at a rate of 19% and 27% in the HIL group and the LIL group, respectively. The difference was not statistically significant, most likely due to the small study group, which is a limitation of this study. Therefore, at this stage, no causal link can be made between irisin and post-transplant diabetes, as the aetiology of PTDM is multifactorial, depending on classic and specific risk factors for the period after organ transplantation. 

Irisin is a myokine, and its level in the blood increases as a result of muscle activity. Assuming that an increase in irisin may be a protective mechanism against hyperglycaemia, in practice, physical exercise should be recommended to all patients after kidney transplantation, with particular emphasis on patients with risk factors for post-transplant diabetes or those with already developing glycaemic disorders. 

Enlarging the study group and extending the follow-up period may allow for more far-reaching conclusions regarding the influence of irisin on glycaemic control and the development of PTDM. Additionally, the inclusion of an intervention element in the form of standardised physical activity will allow us to develop knowledge about the role of irisin in glucose metabolism.

Irisin seems to prevent obesity and insulin resistance in animal experiments [2]. In human subjects, elevated circulating irisin levels were described as associated with a lower risk of insulin resistance in obese adults [8,9]. It was also reported previously that circulating irisin concentration was negatively associated with obesity and insulin resistance and was lower in type 2 diabetes participants [26]; additionally, circulating irisin levels were significantly decreased in obese patients with insulin resistance compared to their controls [27]. In our study, obesity, with BMI > 30, was a factor connected to the significant growth of irisin in patients after KTx. In obese people, higher irisin levels may be the result of greater irisin production in the more developed adipose and muscle tissue or may reflect a compensatory increase in irisin levels to combat obesity and metabolic syndrome to overcome irisin resistance, just as elevated insulin and leptin levels occur in patients with insulin and leptin resistance. 

In our study, irisin levels before transplantation were low in patients with BMI above and below 30; only after the kidney transplantation procedure and the introduction of diabetogenic immunosuppressive drugs did a large difference in irisin levels appear, levels significantly higher in obese patients, which seems to be consistent with the theory of an adaptive increase in irisin levels in patients with metabolic abnormalities associated with obesity [10]. 

In states of advanced obesity, irisin at physiological concentrations is unable to maintain the balance between energy storage and expenditure. In this situation, it is produced not only in muscle but also in adipose tissue [28]. Decreased irisin levels in people with T2DM may seem surprising because these people often are overweight or obese and, therefore, should rather tend to be inclined towards a higher level of myokines. Additionally, these patients develop insulin resistance, which also often correlates with elevated irisin levels. In turn, in non-obese and moderately obese patients with insulin resistance, irisin release from muscles is reduced as a result of muscle resistance to insulin. However, it remains unclear whether low irisin concentrations in T2DM describe a true cause-and-effect relationship.

The large increase in irisin level (in the HIL group) was not connected to pre-transplant HbA1c, glucose, C-peptide, insulin, HOMA index and cholesterol in our study.

Irisin stimulates nitric oxide production, which has been demonstrated in rats, and this may cause arterial relaxation and, as a consequence, lower blood pressure [8]. In our study, an increase in irisin level was not associated with the blood pressure level in patients in the early moments after KTx. Data on the relationship between irisin levels and blood pressure in human studies are conflicting [10,19,29,30,31].

## 4. Materials and Methods

### 4.1. Participants

We analysed irisin serum levels (ISL) in 47 patients without diabetes (M28, F19, mean age 48.5 years) before and after kidney transplantation (KTx) performed in our centre in 2021 and 2022. The study group (*n* = 47) is representative of the entire population of patients who underwent kidney transplantation at our centre (*n* = 232) during the study period.

All patients had end-stage renal failure before transplantation, were on haemodialysis, peritoneal dialysis or were on predialysis with GFR < 10 mL/min. None of the included patients had diabetes before kidney transplantation, and physical activity before and after transplantation was similar in all patients. Inclusion criteria: over 18 years of age, conscious and voluntary consent to participate in the study. Exclusion criteria: no informed consent to participate in this study and/or steroid therapy before transplantation. Only one patient received a kidney transplant from a living donor; the rest received kidney transplants from deceased donors (DBD (donation after brain death)).

### 4.2. Methods

#### 4.2.1. Irisin Serum Level (ISL)

Blood samples (5 mL) were obtained before KTx and at the following time points: 1, 2, 3, 4 and 5 weeks after KTx. 

The serum was separated after centrifugation, mixed with aprotinin (final concentration: 0.6 Unit/1 mL) in Eppendorf microtubes (GenoPlast Biotech S.A., Rokocin, Poland) and stored at −80 °C pending analysis. Quantification of serum irisin was based on competitive enzyme immunoassay kits (Phoenix Pharmaceuticals, Mannheim, Germany). 

Due to post-transplant serum irisin concentration levels (SIL), the entire group was divided into subgroups with an increase in serum irisin concentration (high irisin level—HIL) and without an increase in serum irisin concentration (low irisin level—LIL).

#### 4.2.2. Lipid Parameters, Glycaemia and Other Biochemical Parameters

Lipid parameters were measured by enzymatic methods using an Abbott Anility C analyser (Abbott, IL, USA). HbA1c was determined with the HPLC (high-performance liquid chromatography) method using a HLC 723 G11 analyser (Tosoh, Tokyo, Japan). 

Glycaemic control during the post-transplant period was carried out after fasting (8 AM) and around 1 PM (non-fasting, postprandial), as taken in accordance with our centre’s procedures for the care of organ transplant patients. Some patients stayed in hospital for a shorter period, e.g., nine days; hence, systematic glycaemic monitoring was discontinued on that day. If the patient remained in hospital, measurements up to day 15 were included in the analysis. 

Serum creatinine, glucose, C-peptide, insulin, homocysteine, albumin and uric acid were measured according to standard procedures in the certified laboratory of our university clinical centre directly before transplantation and before administration of immunosuppressive drugs.

#### 4.2.3. Statistical Methodology 

Statistical analysis was performed using the R statistical package (version 3.6.3). 

The baseline characteristics of patients were presented as means, SD (standard deviation) and quartiles for numerical data, whereas qualitative variables were reported as counts and percentages. The differences in between-group baseline characteristics were evaluated using the unpaired *t*-test (or Mann–Whitney U test) for numerical data and the chi-square test for categorical variables. 

A linear mixed-effect model was fitted to glucose level with time, irisin subgroups and time-by-irisin subgroup interactions as fixed effects and random intercepts. Based on this model, mean values for glucose level were estimated with 95% CI for specified measurement points and irisin subgroups. We also estimated mean within-group differences in change from measurement 1 (baseline) to each other measurement. The Kenward–Roger approximation was used to calculate denominator degrees of freedom in the *t*-test.

No formal adjustment for multiple testing was made. The two-tailed tests were carried out at a significant level of 0.05. 

## 5. Conclusions

Irisin levels increase significantly in some patients after kidney transplantation, and this is accompanied by lower blood glucose levels in the early post-transplant period. Whether an increase in irisin levels results in better glycaemic control remains questionable and requires further research, as well as the relationship between irisin levels and the occurrence of PTDM.

## Figures and Tables

**Table 1:** Characteristics of the study group of patients after KTx and comparison of groups with and without an irisin increase (HIL and LIL group).

Variables	LIL Group (*n* = 26)	HIL Group (*n* = 21)	Total (*n* = 47)	*p* Value
Age				0.374
Mean (SD)	49.885 (12.054)	46.667 (12.431)	48.447 (12.197)	
Median (Q1, Q3)	48.500 (39.000, 58.250)	46.000 (37.000, 57.000)	47.000 (38.500, 58.000)	
Gender				0.770
Female	11 (42.3%)	8 (38.1%)	19 (40.4%)	
Male	15 (57.7%)	13 (61.9%)	28 (59.6%)	
BMI kg/m^2^				0.987
Mean (SD)	25.160 (3.518)	25.142 (4.243)	25.152 (3.815)	
Median (Q1, Q3)	24.750 (23.000, 27.750)	24.500 (22.400, 30.000)	24.500 (22.650, 27.800)	
BMI > 25				0.821
<25	14 (53.8%)	12 (57.1%)	26 (55.3%)	
>25	12 (46.2%)	9 (42.9%)	21 (44.7%)	
BMI > 30				0.041
<30	25 (96.2%)	16 (76.2%)	41 (87.2%)	
>30	1 (3.8%)	5 (23.8%)	6 (12.8%)	
Cause of ESRD *n* (%)				0.257
GN	14 (58.3%)	10 (47.6%)	24 (53.3%)	
ADPKD	4 (15.4%)	4 (19.0%)	8 (17.0%)	
IN	0 (0.0%)	2 (9.5%)	2 (4.3%)	
Not known	6 (23.8%)	2 (9.5%)	8 (17.0%)	
HN/IschN	2 (7.7%)	1 (4.8%)	3 (6.4%)	
Other (a/HUS)	0 (0.0%)	2 (9.5%)	2 (9.5%)	
Dialysis modality before KTx *n* (%)				0.488
HD	17 (65.4%)	17 (81.0%)	34 (72.3%)	
PD	5 (19.2%)	2 (9.5%)	7 (14.9%)	
PREE	4 (15.4%)	2 (9.5%)	6 (12.8%)	
Pretransplant dialysis time (months)				0.005
Mean (SD)	26.032 (22.495)	81.494 (92.372)	50.813 (69.005)	
Median (Q1, Q3)	23.623 (5.451, 37.443)	51.672 (16.557, 109.311)	29.377 (10.639, 56.590)	
2nd and 3rd KTx *n* (%)				<0.001
	0 (0.0%)	8 (38.1%)	8 (17.0%)	
AR n (%)				0.466
	0 (0%)	0 (0%)	0 (0%)	
DGF n (%)				0.452
	5 (19.2%)	6 (28.6%)	11 (23.4%)	
Tacrolimus use *n* (%)				0.466
	24 (92.3%)	18 (85.7%)	42 (89.4%)	
Tacrolimus blood concentration (ng/mL) on 6th day after KTx *n* = 42				0.001
Median (Q1, Q3)	12.650 (10.850, 16.000)	9.000 (7.900, 10.900)	11.000 (8.400, 13.000)	
Cyclosporin use *n* (%)				0.466
	2 (7.7%)	3 (14.3%)	5 (10.6%)	
Cyclosporin blood concentration (ng/mL) on 6th day after KTx, *n* = 5				0.84
Mean (SD)	242.625 (40.84)	254.083 (62.47)	249.500 (49.07)	
Median (Q1, Q3)	242.625 (213.750, 271.500)	223.000 (213.250, 326.000)	223.000 (213.750, 271.500)	
Induction use (basiliximab/anti-thymocyte globulin) n (%)				0.108
	23 (88.5%)	21 (100.0%)	44 (93.6%)	
Two weeks prednisone cumulative dose (mg/kg)				0.36
Mean (SD)	4.68 (0.9)	4.97 (1.2)	4.8 (1.1)	
Median (Q1, Q3)	4.430 (4.192, 5.343)	5.021 (3.923, 5.738)	4.459 (3.924, 5.719)	
Duration of hospitalisation after KTx (days)				0.432 *
Median (Q1, Q3)	14.000 (12.000, 18.000)	15.000 (12.000, 27.000)	14.500 (12.000, 18.750)	
Posttransplant global mean blood glucose level (mg/dL)				0.002
Mean (SD)	147.194 (24.522)	127.499 (12.238)	138.003 (21.935)	
Median (Q1, Q3)	144.943 (130.000, 156.198)	125.130 (120.154, 131.500)	131.500 (123.024, 151.262)	
CSC one month after KTx (mg/dL)				0.982
Mean (SD)	1.632 (0.522)	1.635 (0.560)	1.633 (0.533)	
Median (Q1, Q3)	1.615 (1.162, 1.922)	1.665 (1.350, 1.935)	1.640 (1.200, 1.925)	
eGFR 4p MDRD				0.742
Mean (SD)	46.762 (15.830)	48.770 (25.168)	47.635 (20.191)	
Median (Q1, Q3)	42.750 (35.325, 59.200)	43.700 (35.400, 49.400)	42.750 (35.325, 57.525)	
eGFR CKD-EPI (mL/min/1.73 m^2^)				0.589
Mean (SD)	47.881 (15.745)	51.165 (25.060)	49.309 (20.139)	
Median (Q1, Q3)	44.000 (36.150, 61.475)	44.350 (38.700, 50.300)	44.000 (36.150, 59.850)	
WIT (min)				0.848
Mean (SD)	32.885 (8.267)	33.333 (7.479)	33.085 (7.843)	
Median (Q1, Q3)	31.500 (26.500, 36.500)	33.000 (27.000, 37.000)	32.000 (27.000, 37.000)	
CIT (min)				0.055
Mean (SD)	840.077 (416.737)	1116.050 (531.504)	960.065 (484.656)	
Median (Q1, Q3)	795.000 (614.500, 936.750)	1101.000 (666.000, 1420.000)	839.500 (627.000, 1213.250)	
Number of HLA mismatches				0.202
Mean (SD)	2.538 (0.706)	2.952 (1.431)	2.723 (1.097)	
Median (Q1, Q3)	2.500 (2.000, 3.000)	3.000 (2.000, 3.000)	3.000 (2.000, 3.000)	
Charlson Comorbidity Index				0.869
Mean (SD)	2.808 (0.981)	2.762 (0.889)	2.787 (0.931)	
Median (Q1, Q3)	2.500 (2.000, 3.000)	3.000 (2.000, 3.000)	3.000 (2.000, 3.000)	
Donor gender				0.202
F	10 (38.5%)	12 (57.1%)	22 (46.8%)	
M	16 (61.5%)	9 (42.9%)	25 (53.2%)	
Donor age (years)				0.785
Mean (SD)	45.500 (15.943)	46.714 (13.922)	46.043 (14.9)	
Median (Q1, Q3)	47.000 (38.250, 55.750)	48.000 (38.000, 56.000)	47.000 (38.000, 56.000)	
HbA1c before KTx (mmol/mol)				0.454
Mean (SD)	5.345 (0.442)	5.231 (0.483)	5.297 (0.456)	
Median (Q1, Q3)	5.400 (5.100, 5.600)	5.400 (4.975, 5.500)	5.400 (5.000, 5.575)	
HbA1c IFCC before KTx (%)				0.491
Mean (SD)	34.805 (4.800)	33.675 (5.140)	34.329 (4.910)	
Median (Q1, Q3)	35.350 (32.000, 38.000)	35.350 (30.750, 36.850)	35.350 (31.000, 37.750)	
Glycaemia before KTx (mg/dL)				0.679
Mean (SD)	83.333 (7.418)	84.500 (9.619)	83.838 (8.335)	
Median (Q1, Q3)	81.000 (77.000, 87.000)	84.000 (77.750, 91.500)	83.000 (77.000, 91.000)	
Insulin level before KTx (uU)				0.399 *
Median (Q1, Q3)	6.250 (4.325, 7.000)	6.950 (4.000, 9.200)	6.400 (4.125, 7.875)	
HOMA-IR Index before KTx				0.434 *
Median (Q1, Q3)	1.240 (0.800, 1.443)	1.330 (0.952, 2.155)	1.250 (0.871, 1.618)	
HOMA over the normal range before KTx > 2 *n* (%)				0.214
	3 (14.3%)	5 (31.2%)	8 (21.6%)	
QUICKI before KTx				0.681
Mean (SD)	0.374 (0.028)	0.369 (0.045)	0.372 (0.036)	
Median (Q1, Q3)	0.368 (0.360, 0.394)	0.366 (0.340, 0.387)	0.368 (0.354, 0.391)	
QUICKI under the normal range before KTx < 0.34 *n* (%)				0.230
	2 (10.0%)	4 (25.0%)	6 (16.7%)	
Homocysteine before KTx (umol)				0.104
Mean (SD)	32.589 (20.808)	23.439 (8.232)	28.835 (17.268)	
Median (Q1, Q3)	26.200 (19.905, 35.540)	22.900 (18.440, 28.950)	23.510 (19.710, 32.400)	
Cholesterol before KTx (mg/dL)				0.642
Mean (SD)	196.826 (57.728)	189.000 (40.229)	193.615 (50.827)	
Median (Q1, Q3)	188.000 (159.000, 215.500)	189.500 (159.500, 208.500)	188.000 (159.000, 215.500)	
Triglyceride before KTx (mg/dL)				0.437
Mean (SD)	138.696 (47.266)	126.875 (44.659)	133.846 (45.993)	
Median (Q1, Q3)	140.000 (110.000, 154.500)	126.500 (93.750, 165.750)	131.000 (102.000, 157.000)	
HDL cholesterol before KTx (mg/dL)				0.747
Mean (SD)	46.773 (9.492)	47.875 (11.342)	47.237 (10.178)	
Median (Q1, Q3)	49.000 (39.250, 51.750)	48.500 (38.500, 55.500)	49.000 (39.000, 54.250)	
LDL cholesterol before KTx (mg/dL)				0.581
Mean (SD)	128.000 (55.263)	119.250 (35.430)	124.410 (47.777)	
Median (Q1, Q3)	118.000 (91.000, 144.000)	121.500 (100.500, 130.250)	120.000 (91.000, 138.000)	
Uric acid before KTx (mg/dL)				0.302
Mean (SD)	5.582 (1.985)	4.969 (1.454)	5.324 (1.785)	
Median (Q1, Q3)	5.000 (4.075, 7.350)	5.050 (3.950, 5.625)	5.000 (4.000, 7.150)	
C-peptide before KTx (ng/mL)				0.743 *
Median (Q1, Q3)	5.600 (4.710, 7.750)	6.695 (3.942, 7.795)	5.790 (4.540, 7.750)	
Albumin before KTx (g/L)				0.382 *
Median (Q1, Q3)	38.000 (4.600, 39.500)	29.500 (4.000, 39.250)	37.000 (4.300, 39.500)	

* Mann–Whitney U test. ADPKD—autosomal dominant polycystic kidney disease; AR—acute rejection; BMI—body mass index; CIT—cold ischaemia time; CSC—creatinine serum concentration; DGF—delayed graft function; eGFR CKD-EPI—estimated glomerular filtration rate and chronic kidney disease epidemiology collaboration; eGFR 4p MDRD—estimated glomerular filtration rate and 4-point modification of diet in renal disease formula; ESRD—end-stage renal disease; HBA1c—glycosylated hemoglobin, Type A1c; HD—hemodialysis; HDL—high-density lipoprotein; HIL—high irisin level; HLA—human leucocytes antigens; IschN—ischemic nephropathy; HOMA-IR Index—homeostatic model assessment of insulin resistance index; HUS—hemolytic uremic syndrome; KTx—kidney transplantation; LIL—low irisin level; *n* – number of patients; PD—peritoneal dialysis; PREE—preemptive kidney transplantation; QUICKI—quantitative insulin-sensitivity check index; SD—standard deviation; WIT—warm ischaemia time; Q1—the first quartile; Q3—the third quartile.

**Table 2:** Results of the linear mixed-effect model with repeated measurements of fasting blood glucose measurements for between-group differences regarding irisin subgroups (HIL and LIL).

	Patients with Increase in Irisin Concentration HIL Group				Patients with No Increase in Irisin Concentration LIL Group				Between-Group Change	
Days after Transplantation	Number of Patients	Fasting Glucose Serum Level; Mean (95% CI) (mg/dL)	Change from Baseline; Mean (95% CI) (mg/dL)	*p*-Value	Number of Patients	Fasting Glucose Serum Level; Mean (95% CI) (mg/dL)	Change from Baseline; Mean (95% CI) (mg/dL)	*p*-Value	Mean (95% CI) (mg/dL)	*p*-Value
Day 1	7	123 (111 to 135)	-	-	9	150 (139 to 160)	-	-	−26 (−43 to −10)	0.001
Day 2	20	126 (118 to 134)	3 (−19 to 24)	0.999	19	144 (136 to 152)	−6 (−26 to −4)	0.999	−18 (−30 to −7)	0.002
Day 3	19	108 (100 to 117)	−15 (−37 to 7)	0.570	20	120 (112 to 128)	−30 (−49 to −10)	<0.001	−12 (−23 to −0.3)	0.044
Day 4	20	94 (86 to 102)	−29 (−51 to −7)	<0.001	24	110 (103 to 118)	−39 (−59 to −20)	<0.001	−16 (−28 to −5)	0.004
Day 5	19	91 (82 to 99)	−32 (−54 to −10)	<0.001	24	108 (101 to 116)	−41 (−61 to −22)	<0.001	−17 (−29 to −6)	0.003
Day 6	21	95 (87 to 103)	−29 (−50 to −6)	0.001	24	102 (95 to 110)	−47 (−66 to −27)	<0.001	−7 (−18 to 4)	0.187
Day 7	20	96 (88 to 104)	−27 (−49 to −5)	0.002	24	103 (95 to 110)	−47 (−66 to −27)	<0.001	−7 (−18 to 4)	0.223
Day 8	19	95.25 (87 to 104)	−28 (−50 to −6)	0.002	23	100 (93 to 108)	−49 (−69 to −30)	<0.001	−5 (−16 to 6)	0.369
Day 9	19	94 (86 to 103)	−29 (−51 to −7)	<0.001	21	99 (91 to 107)	−50 (−70 to −30)	<0.001	−5 (−17 to 6)	0.384
Day 10	14	96 (87 to 105)	−27 (−50 to −4)	0.006	21	99 (91 to 107)	−51 (−71 to −31)	<0.001	−2 (−15 to 10)	0.688
Day 11	14	94 (85 to 103)	−29 (−52 to −6)	0.002	19	98 (90 to 106)	−52 (−72 to −32)	<0.001	−4 (−16 to 8)	0.540
Day 12	13	95 (85 to 104)	−28 (−52 to −5)	0.004	16	94 (85 to 103)	−56 (−76 to −35)	<0.001	1 (−12 to 13)	0.889
Day 13	10	92 (82 to 103)	−31 (−56 to −6)	0.002	12	97 (87 to 106)	−53 (−75 to −31)	<0.001	−5 (−19 to 10)	0.527
Day 14	9	94 (83 to 105)	−29 (−54 to −4)	0.007	9	99 (88 to 104)	−51 (−74 to −27)	<0.001	−5 (−20 to 11)	0.546
Day 15	7	87 (75 to 99)	−36 (−63 to −10)	<0.001	7	101 (89 to 113)	−49 (−74 to −24)	<0.001	−14 (−31 to 3)	0.110

**Table 3:** Results of the linear mixed-effect model with repeated measurements of postprandial blood glucose measurements for between-group differences regarding irisin subgroups.

	Patients with Increase in Irisin Concentration HIL Group (*n* = 21)				Patients with No Increase in Irisin Concentration LIL Group (*n* = 26)				Between-Group Change	
Days after Kidney Transplantation	Number of Patients	Postprandial Blood Glucose Mean (95% CI) mg/dL	Change from Baseline, Mean (95% CI) mg/dL	*p*-Value	Number of Patients	Postprandial Blood Glucose Mean (95% CI) mg/dL	Change from Baseline, Mean (95% CI) mg/dL	*p*-Value	Mean (95% CI) mg/dL	*p*-Value
Day 1	19	133 (116 to 151)	-	-	19	165 (148 to 182)	-	-	−32 (−56 to −7)	0.012
Day 2	12	140 (119 to 161)	7 (−33 to 47)	0.999	16	173 (154 to 190)	7 (−30 to 44)	0.999	−32 (−60 to −4)	0.026
Day 3	13	143 (123 to 164)	10 (−29 to 49)	0.999	20	168 (151 to 184)	3 (−32 to 38)	0.999	−24 (−51 to 2)	0.069
Day 4	10	148 (125 to 171)	15 (−28 to 58)	0.999	20	170 (153 to 187)	5 (−30 to 40)	0.999	−22 (−50 to 6)	0.131
Day 5	13	135 (115 to 155)	2 (−38 to 41)	0.999	17	166 (148 to 184)	1.42 (−35 to 38)	0.999	−31 (−58 to −4)	0.024
Day 6	14	140 (121 to 160)	7 (−32 to 46)	0.999	16	165 (146.5 to 183)	−0.07 (−37 to 37)	0.999	−24 (−51 to 3)	0.075
Day 7	11	129 (107 to 151)	−4 (−46 to 37)	0.999	21	151 (134 to 167)	−14 (−49 to 20)	0.983	−22 (−49 to 6)	0.123
Day 8	10	136 (113 to 158)	2 (−41 to 45)	0.999	18	152 (134 to 169)	−14 (−49 to 22)	0.993	−16 (−45 to 13)	0.273
Day 9	10	133 (110 to 155)	−1 (−44 to 42)	0.999	16	146 (127 to 164)	−19 (−56 to 18)	0.903	−13 (−42 to 16)	0.373
Day 10	9	138 (114 to 162)	5 (−40 to 49)	0.999	13	159 (139 to 178)	−7 (−45 to 33)	0.999	−21 (−52 to 10)	0.188
Day 11	7	141 (115 to 168)	8 (−41 to 57)	0.999	16	157 (139 to 176)	−8 (−44 to 29)	0.999	−16 (−48 to 16)	0.326
Day 12	7	132 (105 to 158)	−2 (−51 to 47)	0.999	11	160 (138 to 182)	−5 (−47 to 36)	0.999	−28 (−62 to 6)	0.104
Day 13	4	135 (102 to 169)	2 (−59 to 63)	0.999	8	148 (124 to 172)	−17 (−63 to 29)	0.995	−13 (−54 to 29)	0.551
Day 14	4	151 (117 to 184)	17 (−44 to 79)	0.999	6	182 (154 to 209)	17 (−35 to 68)	0.998	−31 (−75 to 13)	0.162
Day 15	3	136 (97 to 174)	2 (−67 to 72)	0.999	2	156 (110 to 202)	−9 (−91 to 73)	0.999	−20 (−80 to 40)	0.505

## Data Availability

The data presented in this study are available on request from the corresponding author.
